# Compression Behavior of Concrete Columns Strengthened with Fiber-Reinforced Inorganic Composites Based on Magnesium Phosphate Cement

**DOI:** 10.3390/ma16031258

**Published:** 2023-02-01

**Authors:** Qihang Zhang, Xin Zhang, Qiaoling Liu

**Affiliations:** 1 School of Civil Engineering, Shandong Jianzhu University, Jinan 250101, China; 2 Key Laboratory of Building Structural Retrofitting and Underground Space Engineering of Ministry of Education, Jinan 250101, China; 3 Engineering Research Institute of Appraisal and Strengthening of Shandong Jianzhu University Co., Ltd., Jinan 250014, China

**Keywords:** magnesium phosphate cement, fiber-reinforced inorganic polymer composites, FRP, concrete column, confinement

## Abstract

Fiber-reinforced polymer (FRP) composites have become attractive for strengthening and repairing deteriorated concrete structures. However, their poor high-temperature resistance and durability in some extreme environments, such as frequent water-vapor erosion and temperature changes, limit their application. Magnesium phosphate cement (MPC) has been used to repair damaged concrete due to its excellent high-temperature resistance and durability. Therefore, this paper aims to study the compressive behavior of concrete columns strengthened with fiber-reinforced inorganic polymer (FRiP) composites based on magnesium phosphate cement so as to evaluate the confinement effect. Twenty-one cylindrical specimens were prepared to examine the axial compressive behavior of carbon-fiber-reinforced inorganic polymer (CFRiP) specimens based on magnesium phosphate cement confined by one to three layers of carbon-fiber fabrics. They are compared with concrete specimens strengthened with epoxy-based FRP and unconfined concrete specimens. The test results show that compared with the unconfined concrete specimen, the strength of the CFRiP-strengthened specimens based on magnesium phosphate increases by 1.69–2.50 times, and their ultimate strain is enlarged by 1.83–3.50 times. The strength and ultimate strain of the CFRiP-strengthened specimens based on magnesium phosphate are approximately 95% and 60% of those of the specimens strengthened with epoxy-based FRP, respectively. A semiempirical model of concrete confined by the CFRiP system based on magnesium phosphate cement is also proposed. The theoretical prediction is finally compared with the experimental results, indicating that the developed model provides a prediction close to the test results.

## 1. Introduction

Many concrete structures experience performance degradation in the long term due to load and environmental erosion, adversely affecting structural safety [1,2,3,4]. In the past few decades, fiber-reinforced polymer (FRP) has been widely used for strengthening and repairing concrete structures due to its high strength, light weight, and convenient construction [5,6]. FRP reinforcement technology realizes the reinforcement of structures by pasting FRP fabrics onto the surface of concrete structures using a polymer matrix, usually epoxy resin. Many studies have reported that employing FRP systems to restrain reinforced concrete columns can not only improve their compressive strength and deformation capacity [7,8,9,10,11] but also have a protective impact on reinforced concrete structures eroded by chloride ions in corrosive environments [12]. However, some shortcomings of FRP systems limit their broader application in the construction field. The bonding performance of the FRP–concrete interface is the key to FRP reinforcement technology [13,14]. The low glass-transition temperature of epoxy resin leads to the poor fire resistance of FRP systems and the release of toxic gases at high temperatures [15,16,17]. In addition, the effects of harsh environments (e.g., freeze–thaw cycles, humid and hot environments, salt erosion, dry–wet cycles, and ultraviolet radiation) may deteriorate the interfacial bonding properties over time [18,19,20,21,22,23,24]. Therefore, although FRP is increasingly used to protect concrete structures, the durability of FRP-protected concrete may still be lacking.

In order to overcome the above problems, researchers have used an inorganic matrix (mainly cement mortar) instead of epoxy resin in fiber-reinforced systems and combined it with the fabric grids. Such inorganic composites are usually called the fabric-reinforced cementitious matrix (FRCM) [25]. Some scholars have proposed several other names based on the type of matrix/fabric and the type of the reinforcement matrix: Textile-reinforced mortar (TRM) [26], fiber-reinforced mortar (FRM) [27], mineral-based composites (MBCs) [28], textile-reinforced concrete (TRC) [29], and fiber-reinforced inorganic polymer composites (FRiPs) [30].

With the in-depth study of the effectiveness of FRCM systems, they have become common in existing structural reinforcement, and some countries have developed the required specifications for FRCM reinforcement technology [31,32]. The typical inorganic matrix of existing FRCM systems is ordinary Portland cement (OPC). Further, alkali-activated, magnesium-oxychloride, and magnesium phosphate cement types have been used as inorganic binders [33,34,35]. The performance of the interfacial bonding between the traditional Portland cement mortar and fabric is poor due to its consistency and particle size, and the debonding failure of the FRCM is common [36]. Alkali-activated materials lead to efflorescence due to the possibility of free alkali dissolution [37]; magnesium-oxychloride cement also has poor water resistance, which limits its use in humid environments [38].

As a new type of green binder, magnesium phosphate cement (MPC) enjoys a broad range of advantages compared with other cementitious materials. The bond between magnesium phosphate cement and old concrete has high strength. Fei et al. reported that the bond strength of MPC mortar was remarkably higher than that of OPC mortar, and it had better volume stability than ordinary Portland cement [39]. Similar to most inorganic cementitious materials, magnesium phosphate cement offers good high-temperature resistance [40]. Zhu et al. [41] used magnesium phosphate cement instead of epoxy resin to strengthen concrete beams. They found that the flexural bearing capacity of the strengthened beams was 47% higher than that of unstrengthened beams after a fire, which confirmed the effectiveness of magnesium phosphate cement in improving the fire resistance of strengthening systems. Moreover, the frost resistance, impermeability, salt-frost resistance, and chemical corrosion resistance of magnesium phosphate cement are often superior to those of ordinary Portland cement and epoxy resin in harsh environments, so magnesium phosphate cement has been widely used to repair damaged concrete [42,43,44,45,46,47,48]. In summary, using magnesium phosphate cement as a binder instead of epoxy resin has considerable potential to improve the high-temperature resistance and durability of FRP-reinforced concrete.

The current research on the application of magnesium phosphate cement as an adhesive to fabricate inorganic composite materials for reinforcing concrete structures is still minimal. To the best of our knowledge, there is no literature investigating the effectiveness of FRiP based on magnesium phosphate confined concrete in detail. In order to develop a FRiP system based on magnesium phosphate that is more resistant to high temperatures and more durable than traditional FRP systems, we first need to evaluate the feasibility of replacing epoxy resin with magnesium phosphate cement for concrete reinforcement. Therefore, the primary purpose of this paper is to analyze the effectiveness of magnesium phosphate–FRiP as a confinement system of concrete. A carbon-fiber-reinforced inorganic polymer (CFRiP) composite made of magnesium phosphate cement and unidirectional carbon fibers is employed to confine concrete columns. The axial compression test results of cylindrical specimens under the confinement of different layers of CFRiP are studied and compared with the response of the specimens confined using an epoxy-based carbon-fiber-reinforced polymer (CFRP) with the same dimensions and similar mechanical properties. Moreover, the traditional data acquisition method and digital image correlation (DIC) are utilized to evaluate the structural performance of the specimens. A simple semiempirical model is also developed to predict the ultimate axial strength and related ultimate axial strain of concrete confined by the CFRiP system based on magnesium phosphate cement.

## 2. Experimental Program

### 2.1. Raw Materials

The raw materials for preparing magnesium phosphate cement included reburned magnesium oxide (M), potassium dihydrogen phosphate (KDP), a composite retarder, and water. The magnesia was purchased from Liaoning Xinrong Mining Group Co., Ltd., Anshan, China, and had an average particle size of 43.07 µm. Guang Dong Xi Long Science Co., Ltd., Shantou, China, provided potassium dihydrogen phosphate with a particle size of 200/75 to 500/25 (mesh/µm). The composite retarder was made in the laboratory, and tap water was used in the experiments. Table 1 lists the primary chemical components of magnesia. The impregnating binder, epoxy resin ZN-700, was provided by Shanghai Zhinuo Decoration Material Co., Ltd., Shanghai, China, and had a tensile strength, elastic modulus, and elongation at break of 48.8 MPa, 3110 MPa, and 1.73%, respectively. The cost of epoxy resin and magnesium phosphate cement was 35 and 8.8 CNY/kg, respectively, and the required amount of magnesium phosphate cement was approximately 3–4 times that of epoxy resin, proving the economic possibility of replacing epoxy resin with magnesium phosphate cement.

### 2.2. Testing Specimens

A total of 21 concrete cylinders with a diameter of 150 mm and a height of 300 mm were prepared and tested to investigate the effects of the binder type and the number of fabric layers on the axial compressive properties of confined concrete. The specimens were divided into seven groups according to the binder type and the number of fabric layers, with three identical specimens in each group. One group was the unconfined specimen, and the FRiP- and FRP-confined specimens were divided into three groups according to the number of wrapped fabric layers. Table 2 presents the details of the specimens. The specimens were named according to the following rules: The letter C represents the unconfined specimen, the letter E indicates the FRP-confined specimens, and the letter M denotes the FRiP-confined specimens; the number following each letter represents the number of fabric layers, and the number after the hyphen (-) indicates the number of specimens in its group.

### 2.3. Specimen Preparation

The cylindrical specimens in this work were all cast from the same commercial concrete batch. The CFRP fabric was pasted on the surface of the samples cured for 28 days in the laboratory environment, and the fiber direction was oriented along the circumferential direction. An overlapping area with a length of 150 mm was set up to prevent the failure of the lap joint. The FRP specimens were prepared using the wet lay-up procedure. First, the concrete surface was cleaned and a layer of epoxy-resin primer was applied. The concrete columns were then wrapped in unidirectional CFRP fabrics impregnated with epoxy resin. Finally, a layer of epoxy-resin adhesive was coated on the surface of the FRP specimen. According to the previous research of our group [49], the mix proportion of the MPC slurry was as follows: The M/KDP molar and water-to-binder ratios were 6.0 and 0.22, respectively, and the composite retarder content was 4.9% of MgO mass. The weighed raw materials were added to a cement mortar mixer according to the above ratios. They were first added to 50% of water and slowly stirred for 1 min; then, the remaining water was added to the mixture and quickly stirred for 3 min. The prepared magnesium phosphate cement slurry was uniformly applied on the surface of the carbon-fiber fabric in one direction with a roller brush for 3 min so that it fully penetrated the fabric. Both sides of the carbon-fiber fabrics should be impregnated with the slurry for the specimens with multi-layer carbon-fiber fabrics. Subsequently, the treated carbon-fiber fabric was attached to the surface of the concrete specimen along the circumferential direction, and an additional FRiP strip with a width of 50 mm was wound at both ends of the column to prevent premature failure of the column end. The FRP-confined specimens were also treated the same way, and the thickness of each layer of the cement matrix was approximately 1 mm. To measure the compressive strength of magnesium phosphate cement, we prepared prism specimens with the dimensions of 40 × 40 × 160 mm^3^. All the specimens were tested for compressive strength after 14 days of curing.

### 2.4. Material Properties

According to ASTM standard D3039 [50], plate tensile tests were conducted on carbon-fiber fabrics. To compare the effects of different binders on the mechanical properties of carbon-fiber fabrics, we tested three specimens: Specimens impregnated with epoxy resin, specimens impregnated with magnesium phosphate cement, and unimpregnated specimens. All the specimens comprised a layer of flat carbon-fiber fabric with the dimensions 250 mm (length) × 25 mm (width) and were cured in a laboratory environment for 7 days.

### 2.5. Instrumentation and Testing Setup

**Figure 1:**
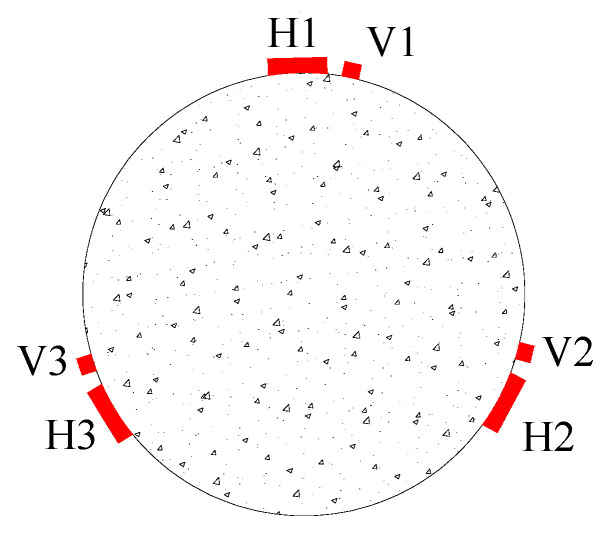
The arrangement of the strain gauges.

Six unidirectional strain gauges were installed at the middle height of each specimen, measuring a length of 50 mm. Three of these strain gauges were used to measure axial strain, and three measured circumferential strain, as shown in Figure 1. The letter H represents the strain gauge used to measure the circumferential deformation of the specimen, and the letter V indicates the axial strain gauge employed to measure the axial deformation of the specimen. All the specimens were tested under concentric compression using a loading machine with a maximum capacity of 3000 kN. Before loading, the end face of the column was polished by an angle grinder so that the axial load could act on the whole section simultaneously. The loading process was controlled by displacement, and the displacement control rate was 0.3 mm/min until the specimen was destroyed.

**Figure 2:**
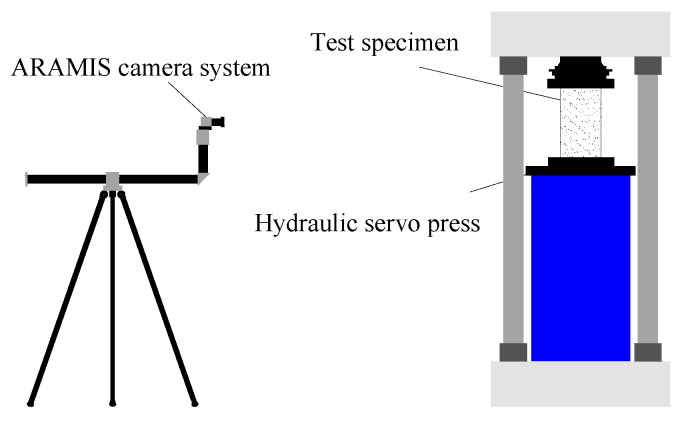
The testing and DIC system setup.

The digital image correlation system monitored the surface deformation of the specimens during loading. The surface of the samples should be covered with artificial spot patterns. To this end, a white primer was sprayed on the surface of the specimen, and then a black marker was used to create randomly distributed spots. The ARAMIS three-dimensional camera system was used for image acquisition. The system was developed by GOM Metrology (Braunschick, Germany). After the test was complete, the collected images were transferred to Gom Correlate 2020 software to calculate the strain. The software was also developed by GOM Metrology. Figure 2 illustrates a schematic of the testing and DIC system setup. A YAW-300E automatic compressive and flexural testing machine measured the compressive strength of the magnesium phosphate cement blocks according to the Chinese national standard, GB/T17671-1999 [51].

## 3. Test Results and Discussion

### 3.1. Failure Mode

**Figure 3:**
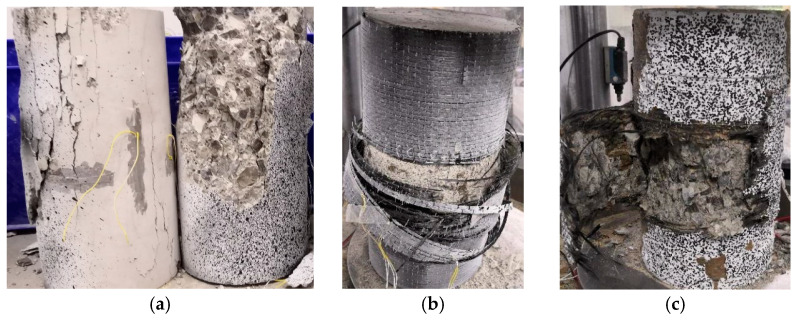
The typical failure modes of the (**a**) unconfined, (**b**) two-layer FRP-confined, and (**c**) three-layer FRiP-confined specimens.

Figure 3 shows the typical failure modes of the unconfined and confined concrete specimens. The unconfined concrete columns show apparent brittleness failure. Firstly, vertical cracks form at the column end. The cracks develop rapidly, and the concrete is crushed as the load increases. Due to the additional confinement at the column end, all the confined concrete specimens are damaged by the CFRP fracture in the middle and high areas of the column. The FRiP-confined specimens do not undergo the debonding failure common in concrete reinforced with the FRCM system. All the confined specimens undergo different degrees of expansion until failure as the load increases. The FRP-confined specimens exhibit a typical failure mode of FRP-confined concrete [52,53]. When the applied load reaches the ultimate load, the CFRP in the middle of the specimen suddenly breaks, and the specimen instantaneously loses its bearing capacity, accompanied by a huge burst sound. The FRiP-confined specimens show two different failure modes. In the case of a large amount of fabric (three-layer and partial two-layer FRiP confinement), the specimens exhibit a failure mode similar to the FRP-confined specimens. However, at a low fabric content (one-layer and two-layer FRiP confinement), the specimens undergo progressive failure, that is, the fibers on the failure surface gradually break, and the bearing capacity drops rapidly, but not as instantaneously as the FRP-confined specimens. This difference may be because the distribution of stress on the fibers is more uniform in thicker FRiP jackets, and more fibers reach ultimate strength when the confinement fails; thus, the confined specimens with a high fabric content experience brittle failure [54]. At a higher fabric content, the concrete core layer of the confined specimen tends to peel more seriously. The main reason is that a higher amount of fabric releases more strain energy, resulting in severer concrete crushing [55].

### 3.2. Flat-Coupon Tensile Tests

**Figure 4:**
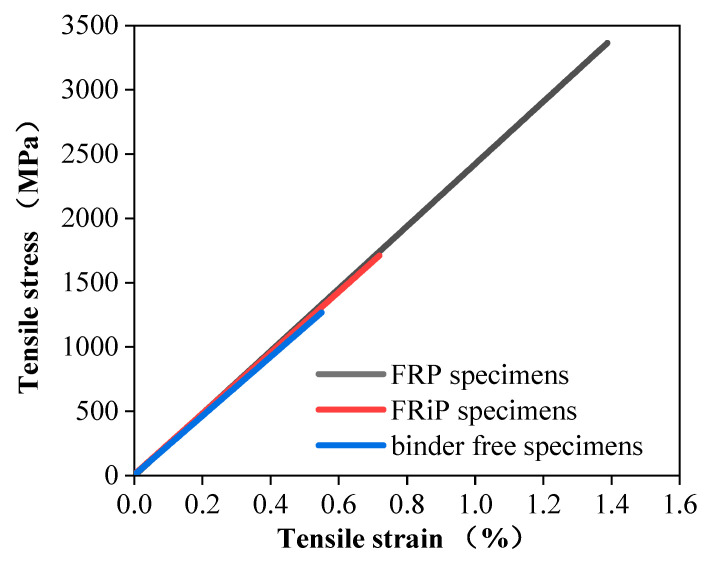
The tensile stress–strain curves of the three types of specimens.

Figure 4 delineates the plate tensile test results of the FRP specimens, FRiP specimens, and binder-free specimens, and Table 3 lists the performance indicators. As shown in Figure 4, the ultimate tensile strength and ultimate tensile strain of the specimens with an inorganic matrix are much lower than those of the samples with an organic matrix and slightly higher than those of the binder-free specimens. Other works have also reported similar results [30,56]. However, the tensile strain curve shows that the CFRiP system based on magnesium phosphate cement used in this work has the same linear elastic constitutive law as the FRP system, and the elastic moduli of the two are approximately similar.

In contrast, the tensile stress–strain curve of the FRCM system appears to be a complex trilinear curve [36], possibly since the FRCM system typically uses web-shaped fibers rather than unidirectional fabric. Previous studies on FRCM systems have shown that the bonding ability between the inorganic matrix and the fiber is low, and the impregnation of the fabric is poor, which causes uneven stress on the fiber bundle [36,57]. Once the mortar cracks, the strain compatibility of the matrix with the fibers disappears quickly, resulting in the premature failure of the FRCM system under tensile loading. The same phenomenon is also observed in this test. The high brittleness of magnesium phosphate cement causes it to be pulled apart prematurely. Some carbon filaments break after the sample loses the binder confinement and is destroyed before reaching the tensile strength of the fiber bundle.

**Figure 5:**
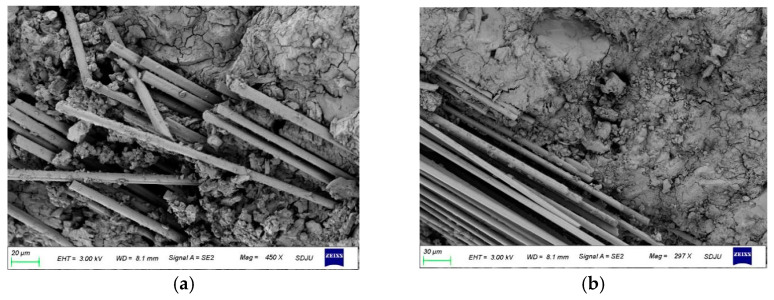
The SEM images of the MPC-based FRiP composites: The (**a**) good and (**b**) poor impregnation of the fibers with the MPC.

Furthermore, the poor impregnation of the fibers with the inorganic matrix is the reason for this phenomenon. Figure 5 shows the typical scanning electron microscopy (SEM) images of the FRiP samples. Figure 5a,b show two cases of good and bad impregnation of the fibers with the inorganic matrix, respectively, which proves that the fibers cannot be impregnated with the inorganic matrix well, inevitably causing the uneven distribution of stress on fiber bundles. This also explains why the tensile strength of the samples with the inorganic matrix is between that of the samples with the organic matrix and the binder-free specimens.

### 3.3. Stress–Strain Behavior

**Figure 6:**
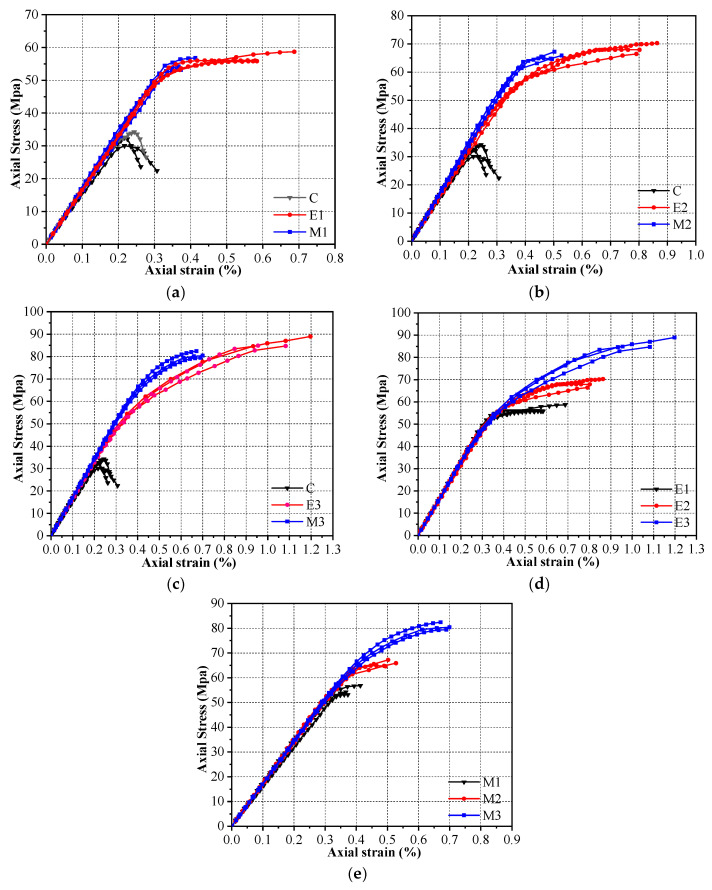
The stress–strain curves of the specimens confined by (**a**) one layer of fabric, (**b**) two layers of fabric, (**c**) three layers of fabric, (**d**) different layers of FRP, and (**e**) different layers of FRiP.

Figure 6 plots the stress–strain curves of the different specimens, representing the relationship between the axial stress and axial strain unless otherwise specified. The axial stress on the specimen is calculated by dividing the axial load by its cross-sectional area. According to the suggestions in some works [58,59], this paper does not use the axial strain data the strain gauges collect. The strain results that the DIC system obtains can often have the same accuracy as those the traditional sensors collect [60,61,62]. Consequently, the axial strain is obtained by dividing the reading of the displacement sensor of the testing machine by the height of each specimen. It was then compared with the strain obtained from the digital image correlation system. The results demonstrate that the two follow the same trend, and the maximum difference in the ultimate strain does not exceed 15%.

Figure 6a–c contrasts the stress–strain curves of the FRP-confined specimens with those of the FRiP-confined specimens with the same number of fabric layers. Figure 6d,e compares the stress–strain curves of two types of confined specimens at various fabric dosages. It is generally believed that the ultimate state of the FRP-confined concrete is marked by the rupture of the FRP fabrics [63,64]. Therefore, all the stress–strain curves in this paper terminate when FRP is ruptured. Figure 6 demonstrates that the stress–strain curves of all the confined specimens are typical bilinear curves. After the monotonically rising initial branch, the strain-hardening stage starts. In a previous study [65], FRP-confined concrete with a strain-softening behavior was considered “inadequately confined”. There is no evident descending branch in the stress–strain curves of all the specimens in this work. Therefore, it can be considered that the CFRiP system based on magnesium phosphate cement can achieve sufficient confinement of concrete columns. Increasing the number of fabric layers enhances the strength and ductility of the FRP- and FRiP-confined specimens. Although the strength of the two is close, the FRP-confined columns offer better ductility with the same amount of fabric.

Additionally, the stiffness of the FRiP-confined specimens increases slightly with an increase in the fabric content, and their degradation rate is lower than that of the FRP-confined columns, which may be due to the contribution of magnesium phosphate cement to the axial compressive strength. Previous studies have reported the contribution of inorganic matrix mortar to the axial strength in FRCM systems [66,67]. The strength of magnesium phosphate cement (70.1 MPa) is higher than that of core concrete, and the thickness of the binder layer is greater than that of epoxy resin. Thus, this effect further improves by increasing the number of fabric layers. Similar to the FRP-confined specimens, the slope of the hardening stage of the FRiP-confined columns increases with the number of fabric layers and is approximately identical at the same amount of fabric. The relevant evidence demonstrates that the restraint stiffness enlarges the slope of the second part of the stress–strain curve of the FRP-confined specimens [68,69]. The confinement stiffness ($K_{l}$) is defined as $\frac{2E_{f}t}{D}$, where $E_{f}$ is the elastic modulus of the FRP sheet, t indicates the thickness of the FRP sheet, and D represents the diameter of the confined concrete. As mentioned above, the binder has a slight effect on the elastic modulus of the composite, so the FRiP- and FRP-confined specimens have similar confinement stiffness, leading to an approximately identical hardening-stage slope.

### 3.4. Ultimate Condition

**Figure 7:**
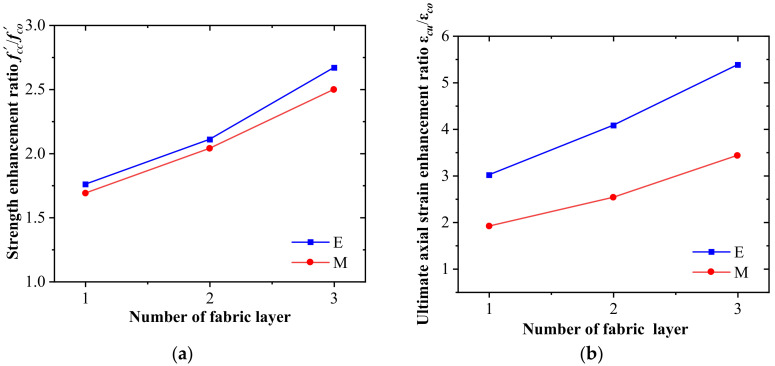
The influence of the number of fabric layers on the (**a**) strength enhancement rate ($f_{cc}^{\prime }f_{co}^{\prime }$) and (**b**) strain enhancement rate (*ε_cu_*/*ε_co_*).

Table 4 summarizes the test results of different restrained specimens: Ultimate axial stress ($f_{cc}^{\prime }$), ultimate axial strain (*ε_cu_*), the strength enhancement rate ($f_{cc}^{\prime }$/$f_{co}^{\prime }$), and the strain enhancement rate (*ε_cu_*/*ε_co_*). The peak stress ($f_{co}^{\prime }$) of the unconfined concrete is considered the average value of three specimens, and the strain (*ε_co_*) corresponding to the peak stress is 0.2%. Figure 7a plots the influence of the number of fabric layers on the strength enhancement rate of the confined specimens. The ultimate axial stress on the confined specimens increases with the number of fabric layers. The ultimate axial stress is the critical parameter of this experimental study determining the confinement effectiveness of the CFRiP system based on magnesium phosphate cement. The strength enhancement ratio ($f_{cc}^{\prime }$/$f_{co}^{\prime }$) of the FRiP-confined specimens ranges from 1.69 to 2.50, which indicates their excellent strength. The strength of the FRiP-confined columns with one to three layers of carbon-fiber fabric is 96.14%, 96.87%, and 93.57% of the corresponding FRP-confined specimens, respectively. This proves that the CFRiP system based on magnesium phosphate cement offers almost the same confinement effect as the epoxy-based FRP system in terms of ultimate strength.

Figure 7b delineates the influence of the number of fabric layers on the strain enhancement rate of the confined columns. Similar to the ultimate axial stress, the ultimate axial strain of the confined specimens increases with the number of fabric layers, and the increase in the ultimate axial strain is more significant than that in the ultimate axial stress. The strain enhancement rate of the FRiP-confined specimens varies from 1.85 to 3.50. Although the deformation capacity of the FRiP-confined concrete significantly improves compared with that of the unconfined concrete, its ultimate axial strain is only approximately 60% of that of the FRP-confined specimens at the same fabric content, which is consistent with previous experimental results indicating that FRP systems are more effective than FRCM systems, especially in strain enhancement [54,56,70]. This phenomenon is primarily attributed to the fact that the epoxy resin matrix has preferable deformability, higher strength, and better impregnation ability than most inorganic matrices, which distributes stress on the fiber bundle more uniformly, thereby offering composite materials with higher strength and deformability [54,71].

### 3.5. Hoop Rupture Strain

This work considers the hoop rupture strain of CFRP to be the average of the measurement results of three hoop strain gauges and DIC equipment. Table 4 tabulates the hoop rupture strain (*ε_h,rup_*) and the corresponding strain efficiency factor (*k_ε_*), i.e., the ratio of the FRP hoop strain to the FRP ultimate tensile strain, of all the specimens. It has been reported that *k_ε_* is often smaller than 1.0, attributed to the uneven deformation of concrete, the local stress concentration of the FRP jacket, the curvature effect of the FRP jacket, and the multiaxial stress state of the FRP jacket [63].

**Figure 8:**
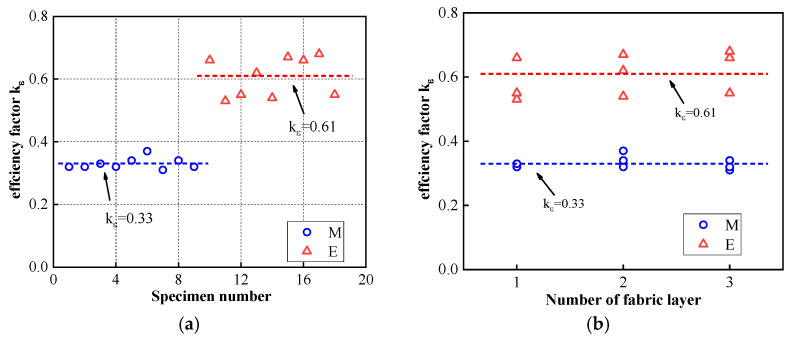
The impacts of (**a**) the binder type and (**b**) the number of fabric layers on the strain efficiency factor, *k_ε_*.

Figure 8a depicts the effect of the binder type on the strain efficiency factor. The strain efficiency factor of the FRiP-confined specimens with a mean value of 0.33 is significantly lower than that of the FRP-confined columns with a mean value of 0.61, which is consistent with the test results of most concrete columns confined by the FRCM system [36,67,72]. The tensile properties of composite materials often determine the effect of structural reinforcement. As mentioned above, the tensile strength and strain of inorganic composite materials are lower than those of organic composite materials, causing the hoop rupture strain of the FRiP-confined columns to be lower than that of the FRP-confined specimens.

Figure 8b depicts the relationship between the strain efficiency factor and the number of fabric layers. It is not difficult to notice that the strain efficiency factor is independent of the number of fabric layers. Nevertheless, some works have reported that the strain efficiency coefficient decreases slightly with an increase in the confinement stiffness [73,74]. This difference may be because the results in the related literature are sourced from an extensive experimental database, while the data in this work are sparse; the matrix type may also impact this difference. Therefore, more research should be conducted on the strain efficiency factor of FriP-confined columns.

### 3.6. Strain Localization

#### 3.6.1. Strain Development on Surface of Specimens

**Figure 9:**
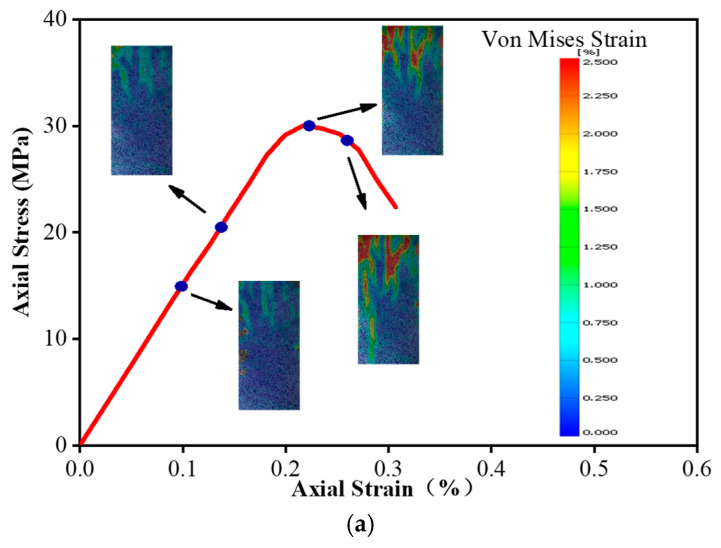

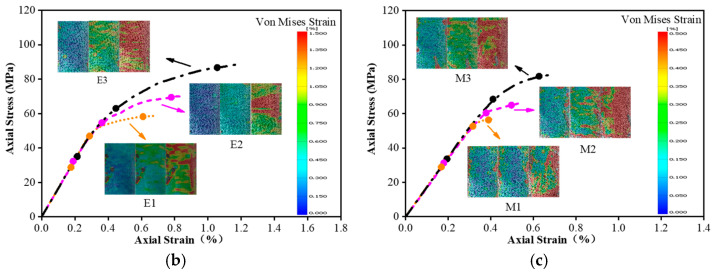
The Von Mises strain evolution of the (**a**) unconfined, (**b**) FRP-confined, and (**c**) FRiP-confined specimens obtained from the digital image correlation.

Using the Von Mises strain data obtained from the DIC system, we can realize the strain localization analysis of the unconfined and confined concrete columns; it should be noted that the analysis results ignore the influence of additional confinement at the column end. The Von Mises strain contours at different stages of the sample are associated with the stress–strain curve to determine the Von Mises evolution result, as drawn in Figure 9.

Figure 9a depicts the Von Mises strain evolution of the unconfined concrete. In the elastic stage, the degree of strain localization on the surface of the sample is low, and the strain distribution is relatively uniform. When the load approaches the peak, the degree of strain localization of the specimen increases significantly. As the stress–strain curve enters the descending stage, the strain localization of the specimen further increases with the development of cracks.

Figure 9b shows the strain localization development of the FRP-confined specimens. The strain localization area of the columns increases with the number of fabric layers, which is consistent with the test results of Pour et al. [60]. According to Pour et al. [68], confinement stiffness is the main factor affecting the strain localization of FRP-confined concrete. Increasing the confinement stiffness expands the strain localization region in FRP-confined concrete specimens. Increasing the number of fabric layers enlarges the confinement stiffness, so the strain localization area of E3 is more extensive than that of E2 and E1, and its deformation is more uniform than that of E2 and E1.

Figure 9c illustrates the strain localization development of the FRiP-confined specimens. The results show the same strain localization development law. The strain localization region of M1 is smaller than that of M2 and M3. Similarly, the lateral confinement of the confined concrete columns reduces the degree of strain localization and makes the strain distribution more uniform compared to the unconfined concrete.

#### 3.6.2. Strain Development along Specimen Height

**Figure 10:**
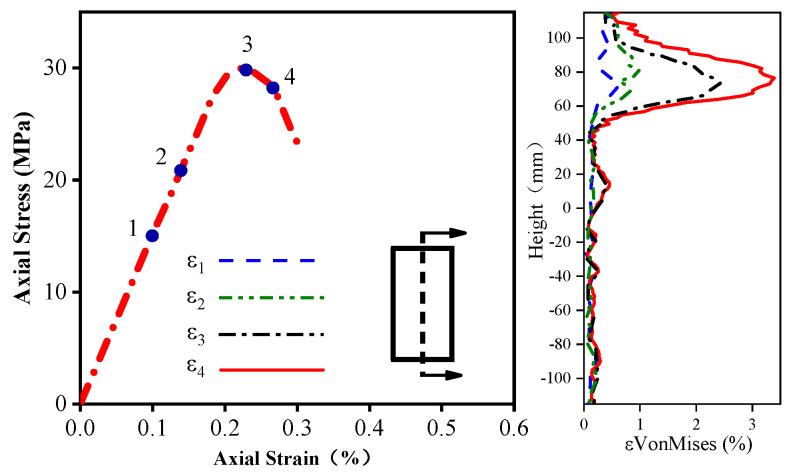
The evolution of the Von Mises strain on the unconfined specimens.

**Figure 11:**
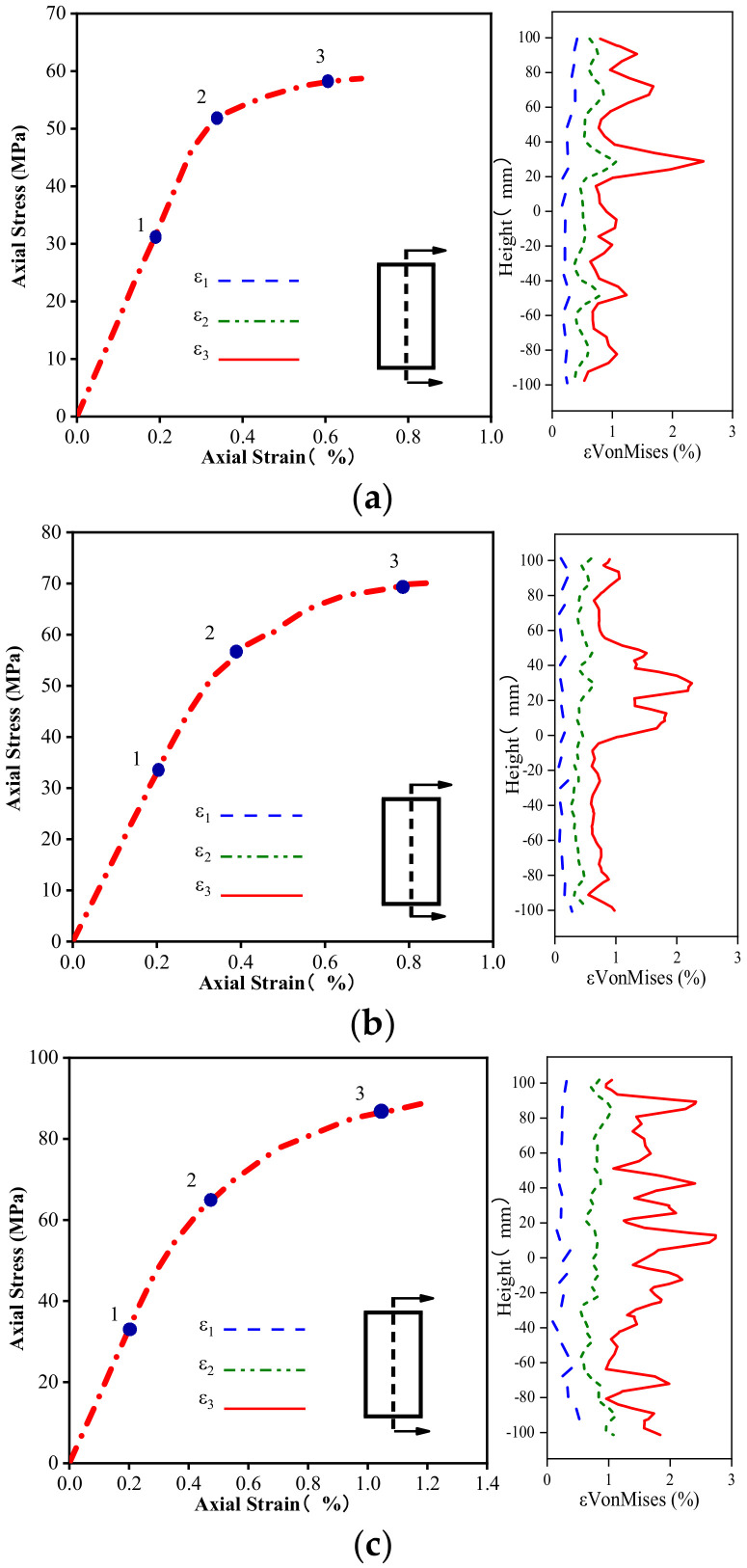
Comparing the Von Mises strain development of (**a**) E1, (**b**) E2, and (**c**) E3.

**Figure 12:**
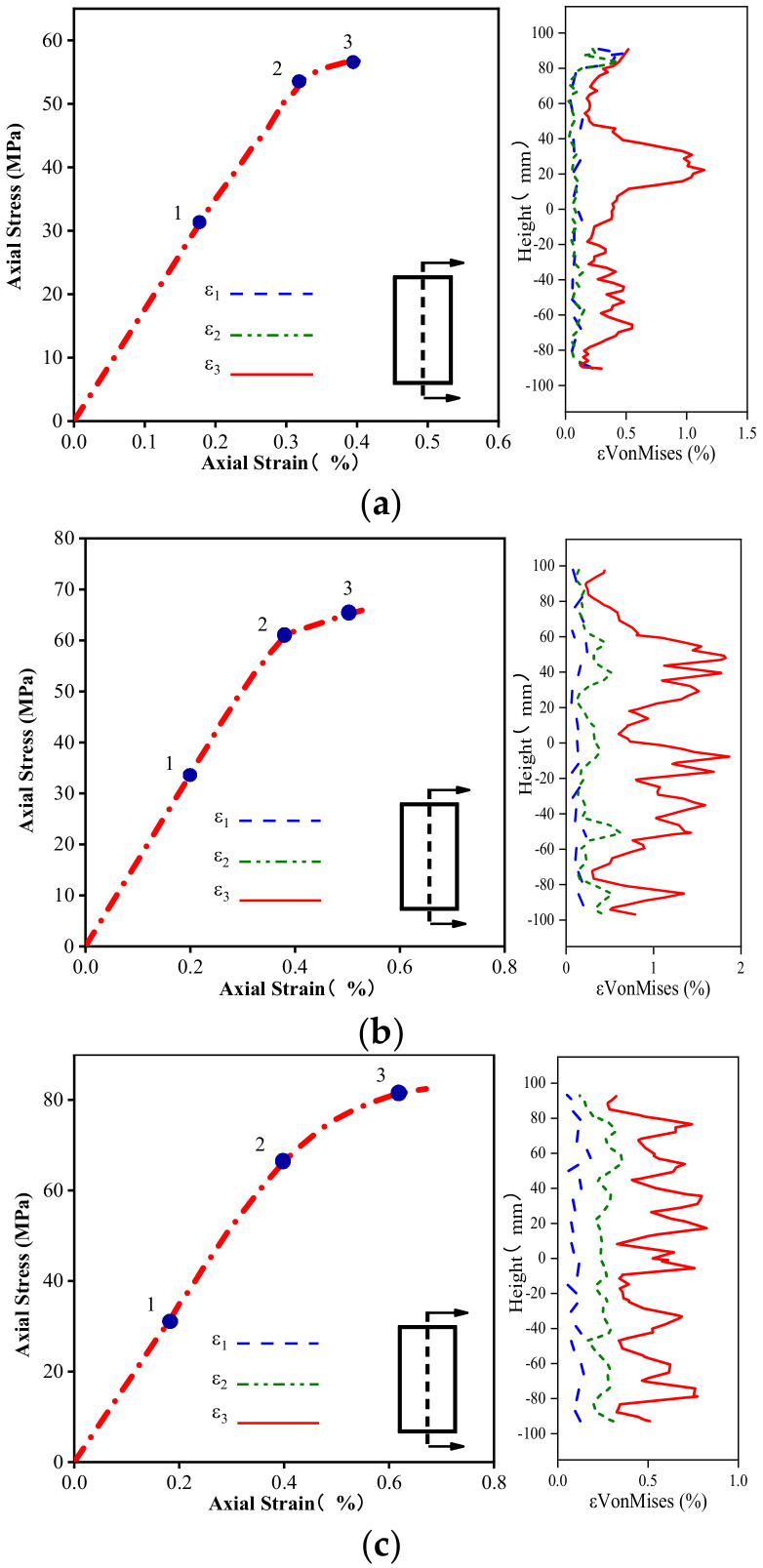
Comparing the Von Mises strain development of (**a**) M1, (**b**) M2, and (**c**) M3.

It is necessary to examine the evolution of the Von Mises strain along the height and horizontal direction of the columns to evaluate their strain localization in more detail. Figure 10, Figure 11 and Figure 12 delineate the evolution of the Von Mises strain along the height of different columns, and the selected section is located at the center of the sample. The height coordinate of zero in the figure indicates that a positive value represents heights higher than the middle position, and a negative value denotes heights lower than the middle position. As shown in Figure 10, the position of the maximum Von Mises strain on the unconfined concrete column is concentrated in the upper part of the specimen, and the rest of the strain does not increase significantly.

Figure 11 and Figure 12 plot the Von Mises strain evolution of the FRP- and FRiP-confined columns, respectively. Both confined specimens reduce the strain localization of concrete to some extent. Under the confinement by the fabric, there is no evident strain localization in the confined concrete specimens when the stress reaches $f_{co}^{\prime }$. When entering the transition section of the two branches of the stress–strain curve, some specimens exhibit the strain localization phenomenon. When the stress approaches $f_{cc}^{\prime }$, the Von Mises strain increases significantly, and the strain localization increases. When the fabric content is low, the maximum Von Mises strain on the confined column is primarily distributed near its middle.

Furthermore, the strain localization area expands, and the strain distribution on the specimen surface becomes more uniform as the fabric content increases, which is attributed to the increasing homogenization of the strain behavior caused by the increased confinement stiffness. It should be noted that the Von Mises strain level of the FRP-confined columns is higher than that of the FRiP-confined specimens, which is due to the weak deformation capacity of the FRiP-confined specimens. Nevertheless, the strain localization on the surface of the FRiP-confined specimens still exhibits a pattern similar to that of the FRP-confined columns, implying that the FRP confinement stiffness is still the main factor determining the localized development of the surface strain of the confined columns.

#### 3.6.3. Strain Development around Specimen Perimeter

**Figure 13:**
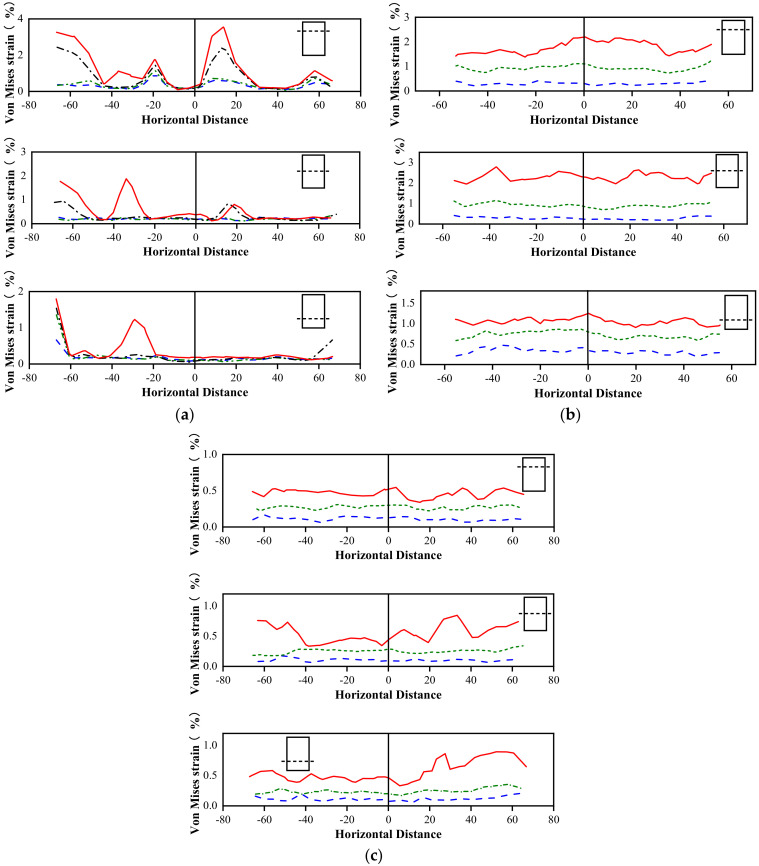
The lateral evolution of the Von Mises strain for the unconfined and confined specimens: (**a**) C; (**b**) E3; (**c**) M3.

The development of the Von Mises strain along the circumferential direction is selected using three different height profiles to study the evolution of the Von Mises strain along the horizontal direction of the columns. This selects the unconfined concrete columns, the FRP-confined columns with three layers of fabric, and the FRiP-confined columns as the representative samples. Figure 13 delineates the evolution of the Von Mises strain along the horizontal direction. Similar to the above comparison results, the Von Mises strain evolution of the unconfined concrete specimens at three different heights shows a high degree of strain localization due to the lack of lateral FRP confinement. In contrast, the FRP- and FRiP-confined columns reduce the degree of strain localization at all heights, making the strain development along the circumferential direction more uniform compared to the unconfined concrete specimens.

Analyzing the strain localization of the confined specimens reveals that the strain distribution is more uniform at a high fabric content, which confirms the inference in Section 3.1 to a certain extent. More fibers reach the ultimate strength in thicker FRiP jackets when the confinement fails, and the failure mode of the FRiP-confined column approaches that of the FRP-confined specimen. However, thicker FRiP jackets still cannot change the strain compatibility of the inorganic matrix with the fibers, so the FRiP-confined columns still cannot achieve higher ductility than the FRP-confined specimens.

## 4. Theoretical Models

### 4.1. Overview

In the past few decades, many scholars have conducted experiments and analyses to study the axial compression behavior of FRP-confined concrete and proposed many models. Among these confinement models for FRP systems, the model proposed by Lam and Teng [75] has been widely recognized for its simplicity and accuracy. This model has been adopted by the American Concrete Institute (ACI) design guidelines (ACI 440.2R-08 2008) [76]. Thus, Teng et al. [77] proposed a more accurate expression for predicting the axial strain and compressive strength of FRP-confined concrete. This improved model has been adopted by Chinese Technical Guideline GB 50608 2010 [78]. Ozbakkaloglu et al. [79] reviewed and evaluated 88 existing models for FRP-confined concrete, affirmed the accuracy of the above two models, and proposed a more accurate confinement model. There are few analytical models for predicting the stress–strain response of FRCM-confined concrete compared with FRP systems. Most existing models for FRCM-confined concrete are primarily derived from the model of FRP-confined concrete, and the new model can be applied to the FRCM system by modifying its parameters. After evaluating several models, Adheem et al. [66] considered that the model of Ombres and Mazzuca [72] and the one proposed in the ACI 549 guideline [31] had better prediction performance than other existing models for FRCM confinement; hence, they proposed a better confinement model based on the model of Ombres and Mazzuca [72].

**Figure 14:**
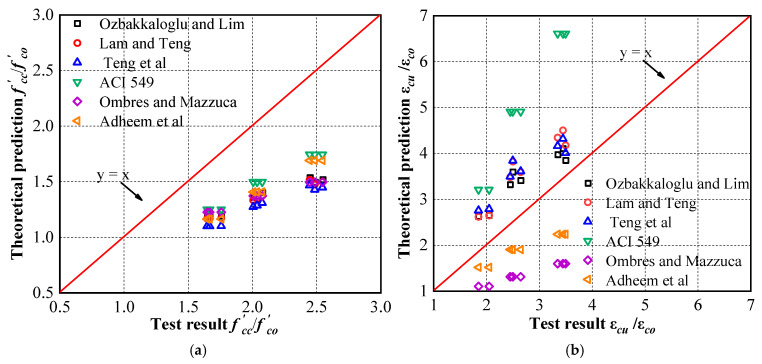
Comparing the performance of the models with the present test results: (**a**) Ultimate axial stress; (**b**) ultimate axial strain.

Figure 14 compares the results predicted by the above model with the test results. The bisector represents the consistency between the experimental and predicted results. The points below the line indicate that the predicted results are conservative, while those above the line represent nonconservative predictions. The results show that all the models predict the ultimate axial stress too conservatively, and the prediction of the ultimate axial strain is either too high or too low. The above high-precision confinement models cannot predict the ultimate axial stress and ultimate axial strain of the specimens well, mainly due to the difference between the confinement mode of concrete in this work and most existing FRP and FRCM systems. These differences primarily originate from the type of cementitious materials and the structure of the fabric. The study of Napoli and Realfonzo [36] also pointed out that the wet lay-up procedure affected the stress–strain behavior of FRCM-confined concrete. Thus, this paper develops a semiempirical confinement model based on the test results of the FRiP-confined specimens and the existing research theories. The accuracy and reliability of the model are also verified by comparing the theoretical prediction with the test results.

### 4.2. Simple Model to Predict Axial Strength and Axial Strain

The ultimate axial stress and ultimate axial strain of confined concrete are usually defined as a function of the actual confining pressure. Equations (1) and (2) express the general form of the model:

(1) $$\frac{f_{cc}^{\prime }}{f_{co}^{\prime }}=1+k_{1}{\left(\frac{f_{l,a}}{f_{co}^{\prime }}\right)}^{\alpha }$$

(2) $$\frac{\varepsilon_{cu}}{\varepsilon_{co}}=c+k_{2}{\left(\frac{f_{l,a}}{f_{co}^{\prime }}\right)}^{\beta }$$

where $k_{1}$, $k_{2}$, α, β, and *c* are empirical constants obtained from optimal fitting analysis to minimize the difference between the experimental and predicted results.

The expression of $f_{l,a}$ in the two equations is as follows:

(3) $$f_{l,a}=\frac{2E_{f}t_{f}\varepsilon_{h,rup}}{D}$$

where $E_{f}$ and $t_{f}$ are the elastic modulus and thickness of the FRiP fabric, respectively; considering that the confinement effect primarily originates from the fiber, the $t_{f}$ value is 0.111 mm for each layer of fabric, *D* indicates the diameter of the confined concrete, and $\varepsilon_{h,rup}$ represents the hoop rupture strain of the FRiP fabric.

It is worth noting that the fabric wrapping form this work uses is close to the FRP system, so the influences of the fiber inclination coefficient and the strain efficiency factor are no longer considered in the definition of $f_{l,a}$, as regarded in most FRCM system models.

Most existing models of FRCM-confined concrete ignore the impact of the inorganic matrix on the performance of confined concrete. Adheem [66] and Napoli [36] pointed out that the inorganic matrix could affect the performance of confined concrete. Adheem [66] also proposed a new dimensionless parameter ($k_{m}$) to quantify the influence of mortar on the performance of confined concrete. The parameter was calculated from 283 experimental and numerical data based on the best-fit analysis and had good reliability. Equation (4) defines $k_{m}$ as:

(4) $$k_{m}=1.7{\left(\frac{4nf_{m}t_{m}}{f_{co}^{\prime }D}\right)}^{0.3}$$

where *n* is the number of the FRCM layers, $f_{m}$ indicates the compressive strength of mortar, and $t_{m}$ represents the thickness of the mortar layer.

This paper introduces $k_{m}$ into the expression of the ultimate strength and ultimate strain by referring to Adheem’s modeling method [66], so Equations (1) and (2) change to the following:

(5) $$\frac{f_{cc}^{\prime }}{f_{co}^{\prime }}=1+k_{1}k_{m}{\left(\frac{f_{l,a}}{f_{co}^{\prime }}\right)}^{\alpha }$$

(6) $$\frac{\varepsilon_{cu}}{\varepsilon_{co}}=c+k_{2}k_{m}{\left(\frac{f_{l,a}}{f_{co}^{\prime }}\right)}^{\beta }$$

Performing best-fit analysis on all test data calculates $k_{1}$ and $k_{2}$ at 1.33 and 17.66, respectively, with an *R*^2^ value of 0.9702 and 0.9711, respectively. Thus, Equations (5) and (6) can be changed into the following:

(7) $$\frac{f_{cc}^{\prime }}{f_{co}^{\prime }}=1+1.33k_{m}{\left(\frac{f_{l,a}}{f_{co}^{\prime }}\right)}^{0.13}$$

(8) $$\frac{\varepsilon_{cu}}{\varepsilon_{co}}=1.75+17.66k_{m}{\left(\frac{f_{l,a}}{f_{co}^{\prime }}\right)}^{1.42}$$

It should be emphasized that according to the description of Eurocode 2 [80], the peak strain (*ε_co_*) and ultimate strain (*ε_cu_*) of unconfined concrete are 0.002 and 0.0035, respectively, and the ratio between the two is 1.75. Therefore, when performing the best-fit analysis, we consider the c value fixed, so the first term at the right hand of Equation (8) equals 1.75. Teng [77] and Wei and Wu [64,81] also adopted this approach in their models.

**Figure 15:**
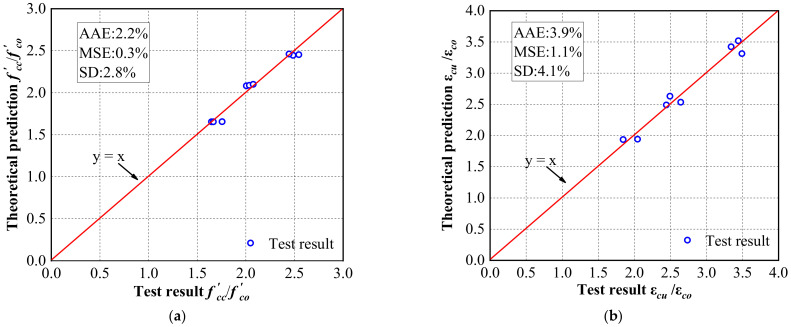
Comparing the predicted (**a**) ultimate axial stress and (**b**) ultimate axial strain with the test results.

Figure 15 compares the model predictions with the experimental results. The average absolute error (*AAE*), the mean squared error (*MSE*), and the standard deviation (*SD*) are also calculated to determine the accuracy of the model. The average absolute error and the mean squared error, defined by Equations (9) and (10), respectively, are used to determine the overall accuracy of the model. Equation (11) also expresses the standard deviation, indicating the degree of change or the magnitude of the prediction spread.

(9) $$AAE=\frac{\sum_{i=1}^{N}\left|\frac{{Test}_{i}-{Theo}_{i}}{{Test}_{i}}\right|}{N}$$

(10) $$MSE=\frac{\sum_{i=1}^{N}{\left({Theo}_{i}-{Test}_{i}\right)}^{2}}{N}$$

(11) $$SD=\sqrt{\frac{\sum_{i=1}^{N}{\left[\frac{{Theo}_{i}}{{Test}_{i}}-{\left(\frac{Theo}{Test}\right)}_{avg}\right]}^{2}}{N-1}}$$

where ${Test}_{i}$ and $Theo_{i}$ are the ith experimental and theoretical values, respectively, *N* indicates the total number of testing columns, and avg represents the sample average.

The points in the figure are closely distributed around the y = x line, indicating that the predicted values of the model are relatively close to the experimental results. Generally, the model reasonably predicts the ultimate strength and ultimate strain of the concrete confined by the CFRiP system based on magnesium phosphate cement. Specifically, the three statistical indicators measuring the accuracy of the model show that the accuracy of the model is lower in predicting the ultimate strain than the ultimate strength, primarily due to the wide variability of the axial strain. It is challenging to establish an accurate model to describe the distribution of strain variation parameters in the confinement mechanism [64]. Table 5 compares the predictions of the developed model with those of the aforementioned typical models. It also lists the mean squared error, average absolute error, and standard deviation of the models. Except for the standard deviation, the statistical indicators of the proposed model are significantly lower than those of the other models, which further implies that the accuracy of the developed model is higher than that of the existing models. It is worth emphasizing that the proposed model is only developed and validated by the experimental data obtained in this study, and further studies are required to verify the accuracy of the model.

## 5. Conclusions

This paper analyzed the effectiveness of the CFRiP system based on magnesium phosphate cement in strengthening concrete columns using axial compression tests and compared them with the columns confined by the epoxy-based FRP system under similar conditions. From the experimental and theoretical results, the following conclusions could be drawn:

- Similar to the FRP-confined specimens, all the FRiP-confined columns failed due to FRP fracture, and no debonding failure occurred. With an increase in the FRP layers, the failure mode of the FRiP-confined specimens changed from progressive failure to sudden failure.
- The CFRiP system based on magnesium phosphate cement had the same linear elastic constitutive law as the FRP system but exhibited lower tensile strength and strain.
- The CFRiP system based on magnesium phosphate cement significantly improved the strength and ductility of the concrete columns. The FRiP-confining system had the same effect as the FRP-confining system on improving the strength of the concrete columns; however, the ultimate strain of the FRiP-confined columns was only 60% of that of the FRP-confined specimens.
- The strain efficiency factor of the CFRiP system based on magnesium phosphate cement was 0.33, much lower than that of the FRP system.
- The DIC results showed that the FRiP- and FRP-confined columns had a similar Von Mises strain evolution law. The uniformity of the strain distribution of the specimens improved as the confinement stiffness increased, proving that the stress distribution was more uniform in thicker FRP jackets, and the failure mode of the FRiP-confined columns was closer to that of the FRP-confined specimens.
- A semiempirical model for predicting the ultimate axial strength and ultimate axial strain of concrete columns confined by the CFRiP system based on magnesium phosphate cement was proposed based on the experimental results. The proposed model was based on limited experimental results, so further studies are required to improve and verify it.

It can be inferred from the test results that the CFRiP system based on magnesium phosphate cement can effectively strengthen concrete columns. However, the poor impregnation of the inorganic matrix and its strain compatibility with fibers limit improving ductility to a certain extent. The improvement in impregnation may be achieved by grinding phosphate and magnesium oxide to reach finer particles, and the improvement in strain compatibility focuses on further enhancing the ductility of magnesium phosphate cement. Thus, developing magnesium phosphate-based inorganic composite materials with higher strength and ductility should be further studied.

## Figures and Tables

**Table 1:** The chemical composition of the dead burned magnesia powders.

Oxide Species	MgO	SiO_2_	CaO	Fe_2_O_3_	Al_2_O_3_
Content (%)	94.81	1.83	1.83	1.06	0.47

**Table 2:** The details of the testing specimens.

Specimen Type	Specimen Group Name	FRP-Type	Number of Fabric Layers	Number of Samples
Unconfined	C	-	-	3
FRP-Confined	E1	CFRP	1	3
FRP-Confined	E2	CFRP	2	3
FRP-Confined	E3	CFRP	3	3
FRP-Confined	M1	CFRiP	1	3
FRP-Confined	M2	CFRiP	2	3
FRP-Confined	M3	CFRiP	3	3

**Table 3:** The material properties of the fibers and FRP composites.

Binder Type	Fiber/FRP Properties		
	Ultimate tensile stress, *f_f_* (MPa)	Ultimate tensile strain, ε*_f_* (%)	Elastic modulus, *E_f_* (GPa)
Epoxy	3365	1.39	242
MPC	1707	0.72	237
-	1262	0.55	229

**Table 4:** The test results of the FRP- and FRiP-confined specimens.

Specimen	$f_{co}^{\prime }$ (MPa)	$f_{cc}^{\prime }$ (MPa)	*ε_cu_* (%)	$f_{cc}^{\prime }$ $/f_{co}^{\prime }$	*ε_cu_/ε_co_*	*ε_h,rup_* (%)	*k_ε_*
E1-1	32.31	58.70	0.65	1.82	3.25	0.92	0.66
E1-2		55.85	0.58	1.73	2.90	0.74	0.53
E1-3		56.04	0.58	1.73	2.90	0.77	0.55
E2-1		66.49	0.79	2.06	3.95	0.86	0.62
E2-2		67.92	0.80	2.10	4.00	0.75	0.54
E2-3		70.30	0.86	2.18	4.30	0.93	0.67
E3-1		88.95	1.20	2.75	6.00	0.92	0.66
E3-2		84.91	1.08	2.63	5.40	0.95	0.68
E3-3		84.90	0.95	2.63	4.75	0.77	0.55
M1-1		53.17	0.37	1.65	1.85	0.45	0.32
M1-2		54.02	0.37	1.67	1.85	0.45	0.32
M1-3		56.82	0.41	1.76	2.05	0.46	0.33
M2-1		65.04	0.49	2.01	2.45	0.45	0.32
M2-2		65.89	0.53	2.04	2.65	0.47	0.34
M2-3		67.23	0.50	2.08	2.50	0.51	0.37
M3-1		80.41	0.70	2.49	3.50	0.43	0.31
M3-2		79.26	0.69	2.45	3.45	0.47	0.34
M3-3		82.44	0.67	2.55	3.35	0.45	0.32

**Table 5:** Comparing the accuracy of the proposed model and classical models.

	Ultimate Axial Stresses $\left(f_{cc}^{\prime }\right)$			Ultimate Axial Strains $\left(\varepsilon_{cu}\right)$		
	AAE	MSE	SD	AAE	MSE	SD
Ozbakkaloglu and Lim	0.34	0.55	0.04	0.30	0.56	0.11
Lam and Teng	0.35	0.59	0.04	0.36	0.86	0.09
Teng et al.	0.38	0.67	0.03	0.37	0.85	0.12
ACI 549	0.13	0.07	0.03	0.94	5.21	0.39
Ombres and Mazzuca	0.33	0.56	0.05	0.48	1.87	0.05
Adheem et al.	0.32	0.45	0.01	0.27	0.68	0.06
Proposed model	0.02	0.003	0.03	0.04	0.01	0.04

## Data Availability

Data are included in the article.
